# Therapeutic Effects of Lavender Oil on Streptozotocin-Induced Diabetes Mellitus and Experimental Thrombosis

**DOI:** 10.3390/antiox14020166

**Published:** 2025-01-30

**Authors:** Valeriu Mihai But, Vasile Rus, Tamás Ilyés, Mădălina Luciana Gherman, Ioana Cristina Stănescu, Sorana D. Bolboacă, Adriana Elena Bulboacă

**Affiliations:** 1 Department of Pathophysiology, “Iuliu Haţieganu” University of Medicine and Pharmacy, Victor Babeş Street, No. 2-4, 400012 Cluj-Napoca, Romania; but.valeriumihai@elearn.umfcluj.ro (V.M.B.); adriana.bulboaca@umfcluj.ro (A.E.B.); 2 Department of Cell Biology, Histology and Embryology, University of Agricultural Sciences and Veterinary Medicine, Calea Mănăștur, No. 3-5, 400374 Cluj-Napoca, Romania; vasile.rus@usamvcluj.ro; 3 Department of Medical Biochemistry, “Iuliu Hațieganu” University of Medicine and Pharmacy, Louis Pasteur Street, No. 6, 400349 Cluj-Napoca, Romania; tamas.ilyes@umfcluj.ro; 4 Experimental Center, “Iuliu Haţieganu” University of Medicine and Pharmacy, Louis Pasteur Street, No. 6, 400349 Cluj-Napoca, Romania; luciana.gherman@umfcluj.ro; 5 Department of Neurology, “Iuliu Hațieganu” University of Medicine and Pharmacy Cluj-Napoca, Victor Babeş Street, No. 43, 400012 Cluj-Napoca, Romania; stanescu.cristina@umfcluj.ro; 6 Department of Medical Informatics and Biostatistics, “Iuliu Haţieganu” University of Medicine and Pharmacy, Louis Pasteur Street, No. 6, 400349 Cluj-Napoca, Romania

**Keywords:** type 1 diabetes mellitus (T1DM), thrombosis, lavender oil (LO), oxidative stress, inflammatory cytokines

## Abstract

Diabetes mellitus is a metabolic disorder associated with oxidative stress, inflammation, and coagulation disturbances, which contribute to microvascular and macrovascular complications. We evaluated the therapeutic effects of lavender oil (*Lavandula angustifolia*) in a streptozotocin (STZ)-induced rat model of type 1 diabetes mellitus (T1DM) with experimentally induced thrombosis. Sixty male Wistar rats were divided into control, thrombosis, diabetes, thrombosis–diabetes, and lavender oil pretreatment groups (100 and 200 mg/kg body weight [bw]). Lavender oil exhibited dose-dependent benefits, with the 200 mg/kg bw dose leading to significant reductions in proinflammatory cytokines (e.g., tumor necrosis factor α (TNF-α); regulated upon activation, normal T cell expressed and secreted (RANTES); and monocyte chemoattractant protein-1 (MCP-1)) and oxidative stress, along with improved glycemic control, the partial restoration of C-peptide levels, and the attenuation of matrix metalloproteinase 2 and 9 (MMP-2 and MMP-9) activity (*p* < 0.0001). Histopathological and coagulation analyses confirmed its organ-protective and antithrombotic effects, including reduced tissue damage, vascular inflammation, and thrombus formation, and prolonged bleeding and clotting times. Our findings suggest that lavender oil exhibits dose-dependent antioxidant, anti-inflammatory, hypoglycemic, and organ-protective effects, indicating its potential as a complementary therapy for managing inflammation in T1DM with or without thrombosis.

## 1. Introduction

Diabetes mellitus is a metabolic disease with major consequences for the health of individuals worldwide [1]. The disease is classified by the American Diabetes Association into four groups according to etiopathogenesis: type 1 diabetes mellitus (T1DM), type 2 diabetes mellitus, gestational diabetes mellitus, and other types. In 2021, approximately 8.4 million individuals worldwide were living with T1DM, with an estimated 500,000 new cases diagnosed annually. The projected life expectancy for a 10-year-old diagnosed with T1DM varies significantly depending on economic context, averaging 13 years in low-income countries compared to 65 years in high-income countries [2]. Patients with T1DM frequently exhibit complications, including cardiac diastolic dysfunction, dyslipidemia, and albuminuria. Notably, endothelial dysfunction has been documented even earlier, during childhood [3]. Type 1 diabetes mellitus is characterized by insufficient or nonexistent insulin synthesis. This is caused by the autoimmune destruction of insulin-producing β-cells in the pancreas, which is mainly caused by the infiltration of macrophages and CD4^+^ and CD8^+^T lymphocytes into the islets [4]. β-cell death is further increased by the production of proinflammatory cytokines and reactive oxygen species (ROS) by innate immune cells, including macrophages, natural killer cells, and neutrophils [5].

Impaired glycemic regulation in patients with diabetes mellitus is associated with hypercholesterolemia, dyslipidemia, and several significant health consequences [6]. Diabetes mellitus is a major cause of microvascular complications including diabetic nephropathy (kidney damage), diabetic neuropathy (nerve damage), and diabetic retinopathy (eye damage). Macrovascular complications associated with diabetes mellitus include atherosclerosis and hemostasis complications (thrombosis). These cardiovascular conditions represent significant causes of premature morbidity and mortality and directly affect organs such as the heart, arteries, and cerebral cortex [5]. Diabetes mellitus causes pathological conditions (oxidative stress, inflammation, platelet hyperactivity, and endothelial dysfunction) that lead to mechanisms that affect platelet activity, the coagulation cascade, or the physiology of the vascular endothelium [6]. The key features of diabetic vasculopathy favoring proinflammatory and prothrombotic disturbances in vascular homeostasis are triggered by endothelial and smooth muscle cell dysfunction. Fluctuations in high glucose levels have been demonstrated to elevate oxidative stress, which produces high levels of glycated proteins and lipids and contributes to endothelial dysfunction. Ineffectively controlled diabetes mellitus, characterized by hyperglycemia, glycemic variability, and elevated levels of free fatty acids, results in elevated ROS levels and a reduction in host antioxidant defense mechanisms. Free fatty acids bind to Toll-like receptors and activate NF-κB, which causes inflammation by elevating the expression of the inflammatory molecules interleukin (IL)-6 and the tumor necrosis factor (TNF)-α [7]. Produced by an increase in fatty acid oxidation, advanced glycated end products (AGEs) are proteins or lipids that are glycated and oxidized via non-enzymatic interactions with glucose or other glycating molecules, including 3-deoxyglucosone, methylglyoxal, and glyoxal. AGE modification harms cells by altering specific protein functions, modifying the extracellular matrix, interacting with matrix receptors on vascular endothelial cells, and binding to advanced glycated end-product receptors (RAGEs) on macrophages and endothelial cells [8]. The activation of these receptors triggers transcription pathways including nuclear factor-κB, which is implicated in inflammatory and immune responses, leading to the upregulation of inducible nitric oxide synthase [9]. Furthermore, RAGE activation in vascular endothelial cells triggers the coagulation pathway and expression of proinflammatory cytokines that stimulate the endothelial tissue factor and chemokine monocyte chemoattractant protein-1 (MCP-1), also known as chemokine CCL2 [8,9]. Moreover, studies indicate that, regulated upon activation, normal T cell-expressed and secreted (RANTES), also known as CCL5, the dominant chemokine in the islet’s inflammatory milieu in prediabetics, can rapidly promote autoimmune diabetes mellitus [10]. Platelets and lymphocytes release RANTES, which increases the adherence of leukocytes to the vascular wall [11].

Experimental models of streptozotocin (STZ)-induced diabetes mellitus associate elevated levels of matrix metalloproteinases 2 (MMP-2) and 9 (MMP-9). These degrade and restructure the extracellular matrix and regulate cytokine production, thereby affecting innate immunity and inflammation [12]. Streptozotocin is an antimicrobial agent that destroys pancreatic islet β-cells often used to induce experimental T1DM [13]. Endothelial cells have been demonstrated to secrete the von Willebrand factor and plasminogen activator inhibitor-1 in response to endothelial cell damage, increasing platelet adhesion and clot formation by inhibiting fibrinolysis [14,15].

Hyperglycemia can exacerbate thrombosis and limit fibrinolysis through several detrimental effects on platelets and fibrin network, both of which are critical contributors to thrombosis [16]. Diabetes-related metabolic alterations have been linked to enhanced platelet hyperactivation and decreased responsiveness to antiplatelet treatments [17]. Platelet hyperactivity also plays an important role in inflammation. Hyperglycemia activates thromboinflammatory responses by enhancing platelet activation and adhesion [18]. Individuals with diabetes mellitus have higher amounts of numerous coagulation factors, which increases susceptibility to the formation of fibrin networks. This is represented by a higher density of the fibrin network and resistance to fibrinolysis. Patients with type 1 and type 2 diabetes mellitus have been found to have plasma levels of fibrinogen, prothrombin, pre-kallikrein, factor V, factor VII, factor VIII, factor X, and factor XI that are much higher than the normal ranges. However, these elevated levels are especially significant in patients whose blood glucose levels are poorly controlled [19].

The management of type 1 diabetes mellitus involves lifelong insulin therapy to regulate blood glucose levels. The development of immunopreventive therapies aims to preserve β-cell function. Non-insulin adjuvant agents are increasingly utilized for their nephroprotective and cardioprotective benefits beyond their glucose-lowering effects [20]. Various traditional medicinal plants have been studied for their protective effects against diabetes that are exerted by modulating oxidative stress and inflammation. Natural therapies may offer additional benefits alongside conventional treatments, potentially improving glycemic control and reducing the complications associated with diabetes mellitus [21].

*Lavandula angustifolia* oil (lavender oil), a mixture of bioactive compounds obtained from a plant species belonging to the *Lamiaceae* and *Lavandula* genera, is clinically useful. Linalool and linalyl acetate, the primary constituents of lavender oil, demonstrated sedative, antidepressant, relaxing, and antiemetic characteristics and exhibited antioxidant and antimicrobial effects, according to previous studies [22,23]. We have demonstrated the benefits of lavender oil (LO) pretreatment in experimentally induced thrombosis [24].

Our study aimed to investigate the effects of lavender oil on an STZ model of diabetic rats with experimentally induced thrombosis, focusing on glycemic levels, oxidative stress parameters, inflammatory cytokines, serum levels of MMP-2 and -9, pancreatic function (C-peptide marker), and histopathological aspects.

## 2. Materials and Methods

Our study received ethics approval from the Ethics Committee of the Iuliu Haţieganu University of Medicine and Pharmacy Cluj-Napoca (AVZ82/29 March 2022) and the National Sanitary Veterinary and Food Safety Authority, Cluj Branch (135/24 May 2022).

### 2.1. Study Design

Sixty 16-week-old male albino Wistar rats obtained from the Animal Facility of the Faculty of Medicine at Iuliu Hațieganu University of Medicine and Pharmacy, Cluj-Napoca, were used. Each rat had a tail length exceeding 13 cm and a body weight ranging from 300 to 400 g. The animals were provided unrestricted access to standard pellet feed (Cantacuzino Institute, Bucharest, Romania) and water throughout this study. Following random allocation into six experimental groups, each comprising ten rats, the animals were housed in polypropylene cages under controlled environmental conditions. These included a 12 h light/dark cycle, a stable temperature of 24 ± 2 °C, and a relative humidity of 60 ± 5%. All of the animals were maintained within the Department of Pathophysiology.

**Figure 1:**
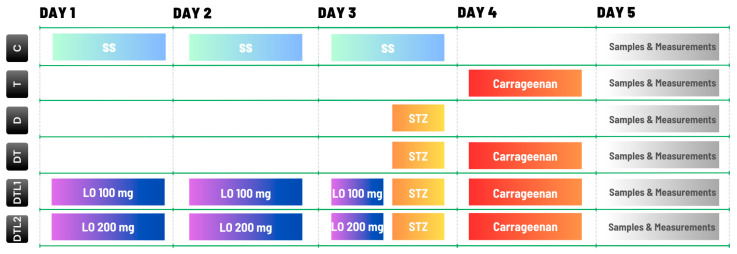
Distribution of experimental groups and treatment protocols: **Control (C)**: Rats in this group are administered intraperitoneal injections of 0.9% saline solution (SS) at a volume of 1 mL throughout the experimental period. **Thrombosis (T)**: Thrombosis is induced on Day 4 through the intravenous injection of 4 mg/kg bw of κ-carrageenan [25]. No additional pretreatments are administered. **Diabetes (D)**: Type 1 diabetes mellitus (T1DM) is induced on Day 3 through the intraperitoneal administration of a single dose of 65 mg/kg bw of streptozotocin (STZ) [26], with no additional interventions provided. **Diabetes–Thrombosis Group (DT)**: T1DM is induced on Day 3 via STZ administration, followed by thrombosis induction on Day 4. **Diabetes–Thrombosis and Lavender Oil 100 mg/kg bw Group (DTL1)**: Rats are pretreated with lavender oil (LO) at a dose of 100 mg/kg body weight (bw), administered intraperitoneally on Days 1, 2, and 3. T1DM is induced on Day 3 with STZ, followed by thrombosis induction on Day 4 with κ-carrageenan. **Diabetes–Thrombosis and Lavender Oil 200 mg/kg bw Group (DTL2)**: Similar to the DTL1 group, these rats receive lavender oil pretreatment but at a higher dose of 200 mg/kg bw intraperitoneally on Days 1, 2, and 3. T1DM is induced on Day 3 with STZ, and thrombosis is induced on Day 4 with κ-carrageenan. On Day 5, all groups undergo sampling and measurements.

All solvents and reactive agents were procured from Sigma-Aldrich, Steinheim, Germany, and possessed analytical-grade purity. The details of the experimental schedule, group assignments, and specific interventions are summarized in Figure 1.

The experimental protocol included a three-day pretreatment phase preceding the induction of thrombosis. On Day 3, T1DM was induced via a single intraperitoneal injection of freshly prepared streptozotocin (65 mg/kg body weight) dissolved in a 0.01 M citrate buffer (pH 4.5). After 48 h, blood samples were collected from the retro-orbital plexus, and glycemia levels were measured using a glucometer (VivaChek Biotech, Hangzhou, China). Rats with glycemic levels ≥ 200 mg/dL were confirmed to have T1DM [22]. To prevent acute complications of T1DM and the associated mortality, two units of an insulin mixture (comprising one rapid-acting insulin and one intermediate-acting insulin) were administered daily at 2:00 p.m.

κ-Carrageenan, a sulfated polygalactan with known proinflammatory properties, was selected as the thrombogenic agent. This compound has been widely used in experimental models to induce pathologies, such as peritonitis, local edema, pleurisy, and thrombosis in laboratory animals. Previously, it was demonstrated that the administration of carrageenan into the dorsal veins of rats induces thrombi [27].

On the fourth day, 4 mg/kg bw of κ-carrageenan dissolved in saline was injected into the tail vein of each rat. Prior to the injection, the tail was ligated 13 cm from the tip to facilitate localized thrombosis. The ligature was maintained for 10 min before removal, following the updated protocol for thrombosis induction, as described by Hagimori et al. This method provides a reliable and reproducible model for studying thrombosis in rats under controlled conditions [25].

Lavander oil, Chemical Abstracts Service (CAS) Number 8000-28-0, was purchased from Sigma-Aldrich, Steinheim, Germany. The certificate of analysis of batch number BCBZ6747 confirms the authenticity of the product. Lavender oil is made by steam distilling freshly cut flowering tops of *Lavandula angustifolia Mill*. (*Lamiaceae*). The distillation product is a pale yellow, amber-tinged liquid with a fresh, sweet, flowery, herbaceous scent and a woody balsamic background. The composition obtained using gas chromatography–mass spectrometry (GC-MS), expressed as %GC mean, was as follows: linalyl acetate, 39.28%; linalool, 27.28%; 4-terpinenol, 2.00%; *(Z)*-β-ocimene, 2.70%; β-caryophyllene, 6.23%; β-farnesene, 3.07%; *(E)*-β-ocimene, 2.36%; *d,l*-borneol, 2.30%; (*R*)- (−)- lavandulol, 1.85%; lavandulyl acetate, 1.76%; α-terpineol, 1.24%; oct-1-en-3-yl acetate, 1.02%; camphor, 0.87%; octan-3-one, 0.82%; 1,8-cineole, 0.78%; geranyl acetate, 0.56%; hexyl butyrate, 0.50% [28].

### 2.2. Measurements

Under light anesthesia induced through the intraperitoneal administration of xylazine (2 mg/kg body weight) and ketamine (20 mg/kg bw), blood samples were collected from the retro-orbital plexus of each rat 24 h after the induction of thrombosis. Samples were drawn into heparinized tubes to prevent coagulation. Plasma was separated through centrifugation at 4 °C for 20 min at 1620× *g*. The collected plasma was placed in Eppendorf tubes and immediately stored at −80 °C to preserve its biochemical integrity until further analysis. At the end of the experimental period, the animals were euthanized using an overdose of anesthetic. All measurements were blinded regarding the group.

#### 2.2.1. Oxidative Stress and Antioxidant Activity

Oxidative stress markers were quantified by measuring the plasma malondialdehyde (MDA) levels as an index of lipid peroxidation [29], NOx [30], and total oxidative capacity (TOS) [31]. The antioxidant activity in the blood was assessed by evaluating the thiol group concentration [32] and total antioxidant capacity (TAC) [33]. The measurements were conducted using a Jasco V-350 UV-VIS spectrophotometer (Jasco International Co., Ltd., Tokyo, Japan).

#### 2.2.2. Inflammatory and Pancreatic Biomarkers

The plasma concentrations of proinflammatory cytokines, including tumor necrosis factor-alpha (TNF-α), regulated upon activation normal T cell expressed and secreted (RANTES), and monocyte chemoattractant protein-1 (MCP-1), were quantified using enzyme-linked immunosorbent assay (ELISA) kits. C-peptide levels, which serve as markers of pancreatic function, were also determined using ELISA. In addition, the levels of MMP-2 and MMP-9 were measured using specific ELISA kits to evaluate tissue remodeling. All of the ELISA measurements were performed using the TECAN SUNRISE absorbance reader, and the intra- and inter-assay coefficients of variation were under 10%.

#### 2.2.3. Coagulation Parameters

The bleeding and clotting times were measured using a stopwatch, and all of the assessments were performed by the same investigator to minimize variability.

#### 2.2.4. Renal and Hepatic Function Tests

Renal function was evaluated by measuring the plasma creatinine and urea levels, while liver function was assessed by measuring the activities of alanine aminotransferase (ALT) and aspartate aminotransferase (AST). These parameters were determined using a Jasco V-350 UV-VIS spectrophotometer (Jasco International Co., Ltd., Tokyo, Japan).

#### 2.2.5. Pancreatic Metabolic Function and Glycemia

Glycemia levels were measured after a 12 h fasting period to assess pancreatic and carbohydrate metabolism. Glycemia serves as a key marker for evaluating the effect of experimental diabetes mellitus on metabolic pathways.

#### 2.2.6. Histological Assessment

Microscopic tissue analysis was performed on 4 mm thick sections obtained from the liver, kidneys, pancreas, and skin. The specimens were fixed in a 10% formalin solution for five days, dehydrated in ethyl alcohol, clarified in 1-butanol, and finally embedded in paraffin. Sections of 5 µm thickness were prepared from the paraffin blocks and then colored using the Goldner trichrome method.

### 2.3. Statistical Methods

The plasma levels of the analyzed biomarkers are expressed as the median [Q1 to Q3], where Q1 and Q3 represent the 25th and 75th percentiles, along with the range {min to max}, where “min” indicates the smallest observed value, and “max” indicates the largest. This method was employed to outline both the central tendency and variability of the data, particularly considering the limited sample size of each experimental group. The nonparametric Kruskal–Wallis test was used to evaluate the overall significance across groups. When this test identified significant differences, pairwise comparisons were performed using post hoc analysis to determine specific intergroup variations. Statistical significance was defined as a *p*-value less than 0.05, and all tests were two-tailed. The variability and distribution of the markers are visually represented using box-and-whisker plots to provide clear graphical summaries of the data. Descriptive statistics and graphical representations were created using Microsoft Office Excel 365, and inferential statistical analyses were performed using Statistica software (version 13.5; StatSoft, Tulsa, OK, USA).

## 3. Results

All of the animals included in this study were analyzed (10 rats in each group). None of the rats died before the completion of the experiment. Type 1 diabetes mellitus and thrombosis were successfully induced.

### 3.1. Oxidative Stress Parameters

**Figure 2:**
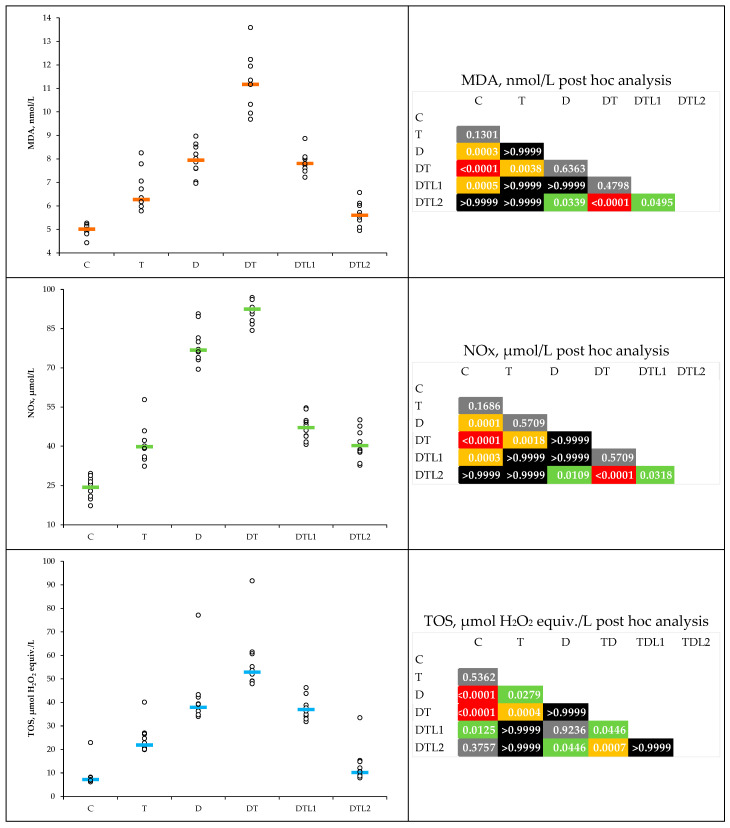
Plasma levels of oxidative stress markers by group (C = control group; T = thrombosis group; D = type 1 diabetes mellitus (T1DM) group; DT = T1DM mellitus and Thrombosis group; DTL1 = DT with lavender oil 100 mg/kg bw pretreatment; DTL2 = (T1DM) with lavender oil 200 mg/kg bw pretreatment).

Thrombosis, TIDM, and their combination led to an increase in the plasma levels of the evaluated oxidative stress markers (Table 1), but the increases did not reach statistical significance at any point (Figure 2). Lavender oil pretreatment (DTL1 and DTL2 groups) reduced the oxidative stress parameters, with DTL2 (200 mg/kg bw) showing significant effects on all of the parameters. The DTL2 group showed higher MDA levels than those in the 100 mg LO-pretreated group. DTL1 displayed significantly reduced NOx levels compared to the DT group (Table 1 and Figure 2).

**Figure 3:**
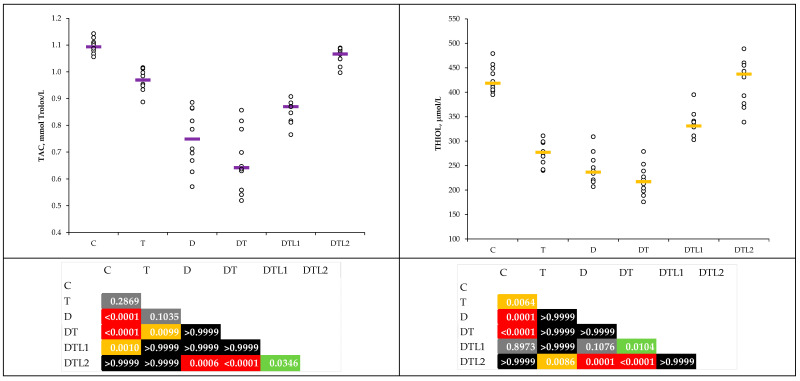
Plasma total antioxidant capacity (TAC) and THIOL levels by group (C = control group; T = thrombosis group; D = type 1 diabetes mellitus (T1DM) group; DT = T1DM mellitus and thrombosis group; DTL1 = DT with 100 mg/kg bw lavender oil pretreatment; DTL2 = DT with 200 mg/kg bw lavender oil pretreatment).

The measured levels of TAC were lower in the T, D, and DT groups than in the control group, while the values of THIOL were lower in T, D, DT, and DTL1 groups (Table 2). Lavender oil pretreatment mitigated the TAC reduction, with values closer to those of the C group for DTL2 (Figure 3). The THIOL levels in both LO pretreatment groups were significantly ameliorated (Table 2 and Figure 3).

### 3.2. Inflammatory Cytokines

**Figure 4:**
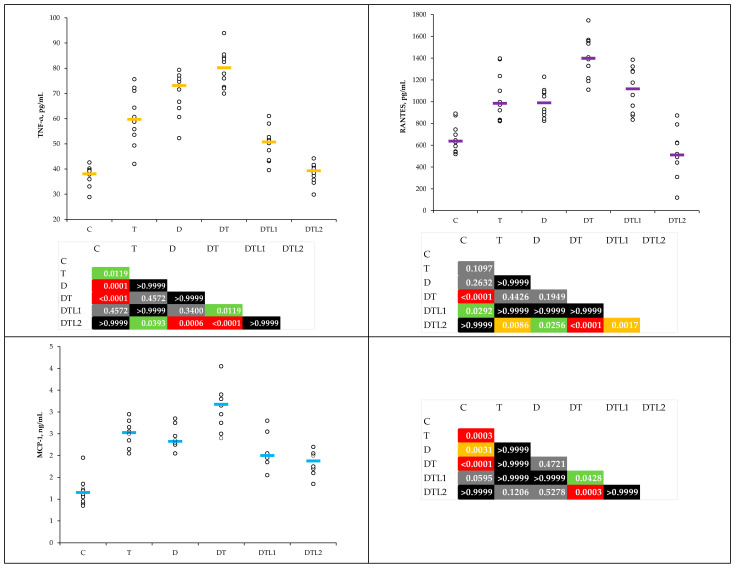
Plasma levels of measured inflammatory cytokines by group: TNF-α and RANTES (C = control group; T = thrombosis group; D = type 1 diabetes (T1DM) mellitus group; DT = T1DM mellitus and thrombosis group; DTL1 = DT with 100 mg/kg bw lavender oil pretreatment; DTL2 = DT with 200 mg/kg bw lavender oil pretreatment; TNF-α = tumor necrosis factor α; and RANTES = regulated on activation, normal T cell expressed and secreted). Continuation. Plasma levels of measured inflammatory cytokines by group: MCP-1 (C = control group; T = thrombosis group; D = type 1 diabetes (T1DM) mellitus group; DT = T1DM mellitus and thrombosis group; DTL1 = DT with 100 mg/kg bw lavender oil pretreatment; DTL2 = DT with 200 mg/kg bw lavender oil pretreatment; and MCP-1 = monocyte chemoattractant protein 1).

The plasma levels of the inflammatory cytokines TNF-α, RANTES, and MCP-1 demonstrated statistically significant differences between the groups (Table 3). Lavender oil pretreatment (DTL1 and DTL2) significantly lowered cytokine levels, with DTL2 levels near or below those of the control group (Table 3 and Figure 4).

### 3.3. Glycemia and C-Peptide Levels

**Figure 5:**
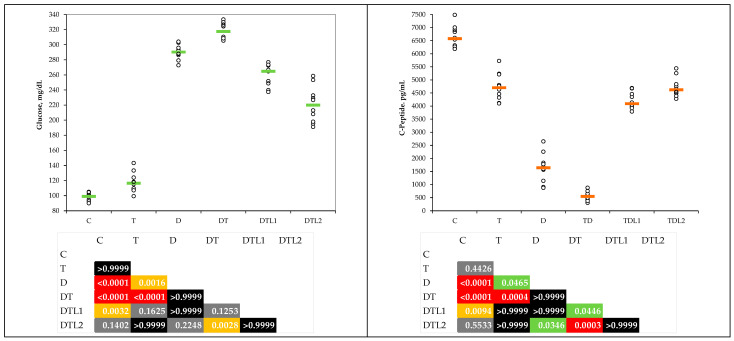
Glycemia and C-peptide level by group (C = control group; T = thrombosis group; D = type 1 diabetes (T1DM) mellitus group; DT = T1DM and thrombosis group; DTL1 = DT with 100 mg/kg bw lavender oil pretreatment; and DTL2 = DT with 200 mg/kg bw lavender oil pretreatment).

The glycemia and C-peptide levels varied among the groups. The type 1 diabetes mellitus (D) and combined thrombosis and T1DM (DT) groups demonstrated significantly increased glycemia levels and substantially decreased C-peptide levels compared with the control group. Lavender oil pretreatment reduced glycemia levels, with significant values in the DTL2 group, and partially restored C-peptide levels, with the higher dosage producing more substantial enhancements but showing no statistically significant difference when compared with the control group (Table 4 and Figure 5).

### 3.4. Metalloproteinases 2 and 9

**Figure 6:**
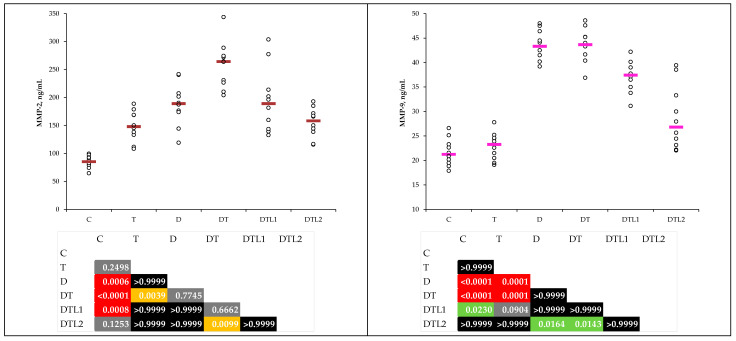
Metalloproteinases 2 and 9 by group (C = control group; T = thrombosis group; D = type 1 diabetes mellitus (T1DM) group; DT = T1DM and thrombosis group; DTL1 = DT with 100 mg/kg bw lavender oil pretreatment; DTL2 = DT with 200 mg/kg bw lavender oil pretreatment; MMP 2 = metalloproteinase 2; and MMP 9 = metalloproteinase 9).

The DT group exhibited the highest levels of MMP-2 and MMP-9, indicating severe matrix remodeling and inflammatory processes (Table 5). Pretreatment with lavender oil reduced both MMP-2 and MMP-9 levels, but significant improvements (*p* < 0.05) were observed only in the DTL2 group (200 mg/kg bw) compared to those in the DT (MMP-2) and D and DT groups (MMP-9), demonstrating a more pronounced effect (Table 5 and Figure 6).

### 3.5. Bleeding and Clotting Time

**Figure 7:**
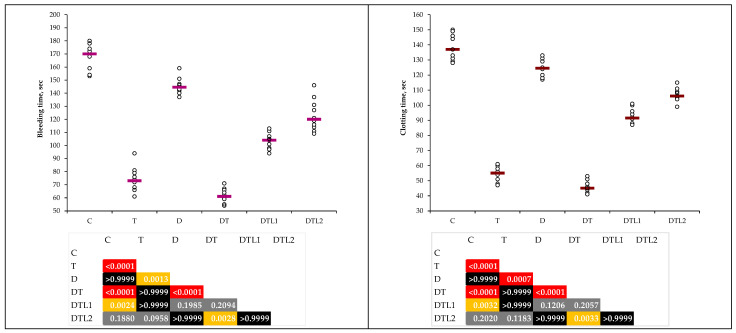
Bleeding and clotting time by group (C = control group; T = thrombosis group; D = type 1 diabetes mellitus (T1DM) group; DT = T1DM and thrombosis group; DTL1 = DT with 100 mg/kg bw lavender oil pretreatment; and DTL2 = DT with 200 mg/kg bw lavender oil pretreatment).

The bleeding and clotting times were significantly shorter in the thrombosis and combined thrombosis and T1DM groups than in the C group. Pretreatment with lavender oil extended the bleeding and clotting times to 200 mg/bw dose (DTL2), showing effects like those in the control group. The DTL2 group showed statistically significant improvements compared with the DT group (Table 6 and Figure 7).

### 3.6. Renal and Liver Functions

The plasma concentrations of creatinine, urea, ALT, and AST were significantly altered in the pathological groups. The combined condition (DT) exhibited the most pronounced dysfunction, characterized by significantly increased levels of creatinine, urea, ALT, and AST compared to the control and thrombosis groups. Double-dose lavender oil pretreatment significantly enhanced renal and hepatic function markers (Table 7).

### 3.7. Histological Assay

Histopathological examination showed no structural changes in the organs examined in the control group (C).

**Figure 8:**
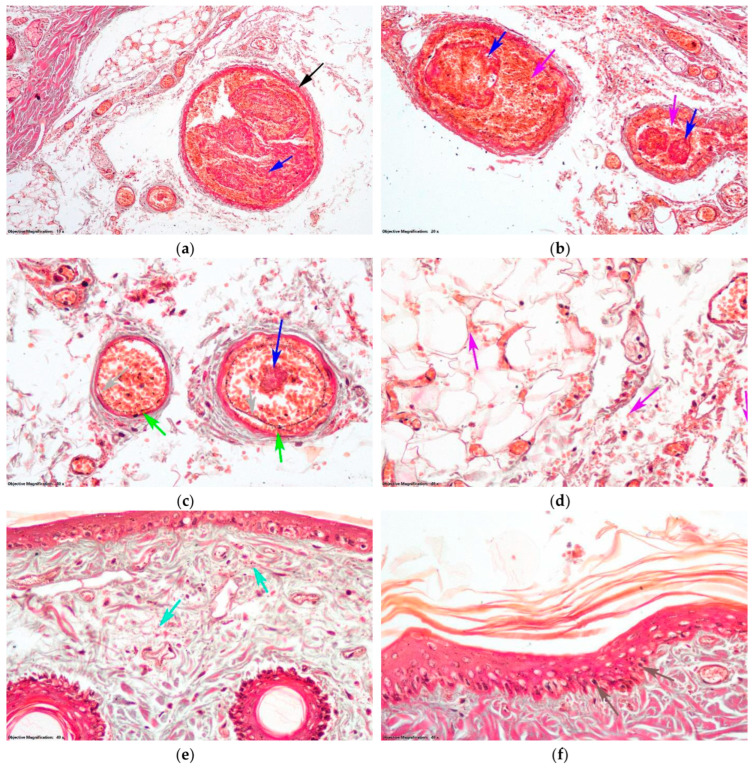
Histological changes (Goldner trichrome staining) in the group with thrombosis: (**a**) Coccygeal artery > black arrow—coccygeal artery; blue arrow—thrombus. (**b**) Medium- and small-sized blood vessels with thrombi from hypodermal structure: magenta arrow—lumen; blue arrow—thrombus. (**c**) Hypodermal blood vessels with vascular dissection: gray arrow—intima; blue arrow—thrombus; green arrow—vascular dissection. (**d**) Hypodermal hemorrhage: purple arrow—extravasated hemocytes. Continuation. Histological changes (Goldner trichrome staining) in the group with thrombosis. (**e**) Edema in the dermis: turquoise arrow—protein granules. (**f**) Keratinocytes in the deep layers of the epidermis entering cell death: brown arrow—pycnotic nuclei.

In the T group, many pathological features were observed in the skin of the tail. The thrombi did not obliterate the lumen of the coccygeal artery (Figure 8a). The thrombi of various sizes that did not occlude the lumen were also present in other medium and small blood vessels in the hypodermis (Figure 8b). Specific aspects of vascular dissection exist in small arteries of the hypodermis. Thus, between the intima and media, there were narrower or wider areas filled with hemorrhages (Figure 8c). Numerous hemorrhages were observed in the hypodermis, in both the connective tissue and adipose panicles (Figure 8d). Edema can be observed in the dermis, especially in the superficial dermis, which increases the distance between collagen fibers. Numerous acidophilic granules, most likely extravasated proteins, were observed in the underlying connective tissue (Figure 8e). In areas where papillary dermal edema is pronounced in the epidermis, especially in the basal layer, and in the first rows of cells in the spinous layer, keratinocyte nuclei are hyperchromic, suggesting premature apoptosis. Additionally, the keratin layer was significantly thinner than that in the control group (Figure 8f).

**Figure 9:**
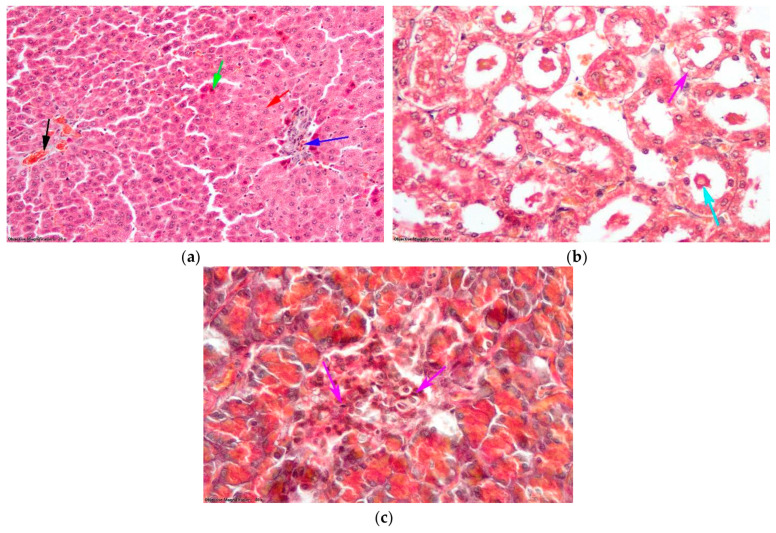
Histological changes (Goldner trichrome staining) in the group with D: (**a**) Hepatic lobule: black arrow—centrilobular venule; blue arrow—portobiliary space; green arrow—hepatocytes in cell death; red arrow—hepatic steatosis with small droplets. (**b**) Nephron tubules: magenta arrow—nephrocytes with degeneration; turquoise arrow—cellular detritus in the lumen of the tubules; gray arrow—hyaline thrombi. (**c**) Islet of Langerhans: purple arrow—endocrine cells undergoing cell death.

In group D, hepatocytes located in the outer third of the liver lobule exhibited small-droplet steatosis. Additionally, some of these hepatocytes were in the early stages of cell death, as shown by their intense acid-fast-stained cytoplasm (Figure 9a). The kidneys exhibit localized areas of granule-vacuolar degeneration in the nephrocytes of the proximal tubule together with cellular debris present in the tubule lumen. Intermittent hyaline thrombi, which did not completely block the tubules, were visible on the surface of the section (Figure 9b). The presence of necrosis in the islets of Langerhans cells in the pancreas indicates the induction of T1DM (Figure 9c).

**Figure 10:**
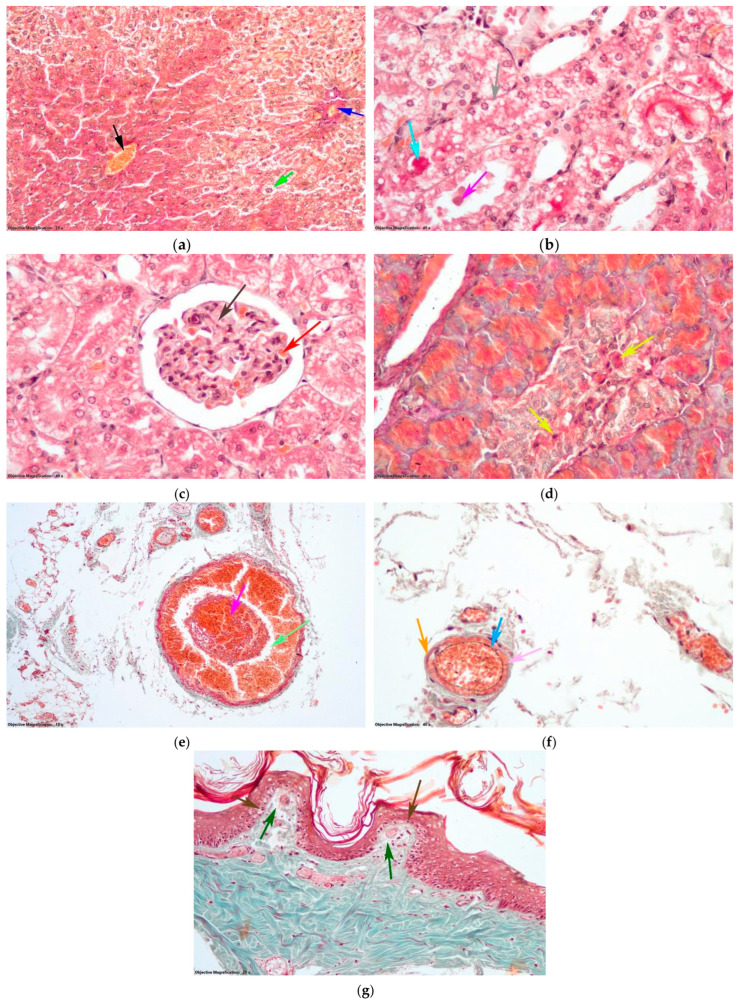
Histological changes (Goldner trichrome staining) in the group with D and thrombosis (DT group): (**a**) Hepatic lobule: black arrow—centrilobular venule; blue arrow—portobiliary space; green arrow—hepatic steatosis with large droplets. (**b**) Nephron tubules: gray arrow—vacuolar degeneration; purple arrow—cellular detritus in the lumen of the nephrons; turquoise arrow—hyaline thrombi. (**c**) Renal corpus: brown arrow—mesangial hyperplasia; red arrow—capillary. (**d**) Endocrine pancreas: yellow arrow—endocrine cells from the islet of Langerhans undergoing cell death. (**e**) Coccygeal artery: magenta arrow—thrombus; mint arrow—lumen of the artery. (**f**) Arteriole from hypodermis: light blue arrow—intima; orange arrow—medial muscle cell; pink arrow—vascular dissection. Continuation. (**g**) Dermis and epidermis: dark green arrow—edema in papillary dermis; maroon arrow—pycnotic nuclei of keratinocytes.

In the DT group, hepatocytes located in the outer third of the hepatic lobules displayed hepatic steatosis, which was characterized by the presence of large droplets (Figure 10a). Within the kidneys, certain nephrocytes located in proximal tubules exhibited vacuolar degeneration. In addition, the lumen of these nephrocytes contained cellular debris and hyalinosis, as shown in Figure 10b. In a few glomeruli, slight hyperplasia of the mesangium was observed (Figure 10c). In the pancreas, cells of the islets of Langerhans showed necrosis (Figure 10d). In the skin, the aspects were similar to those of group T, with thrombi that did not obliterate the lumens of the large vessels (Figure 10e) and intimal dissection in the four medium and small arteries (Figure 10f). Hemorrhage was present in the adipose panicles of the hypodermis, as well as in the deep dermis. In the superficial dermis, there was no hemorrhage, only in places where edema was pronounced. In these areas, the keratinocyte nuclei in the deep layers of the epidermis were pycnotic (Figure 10g).

**Figure 11:**
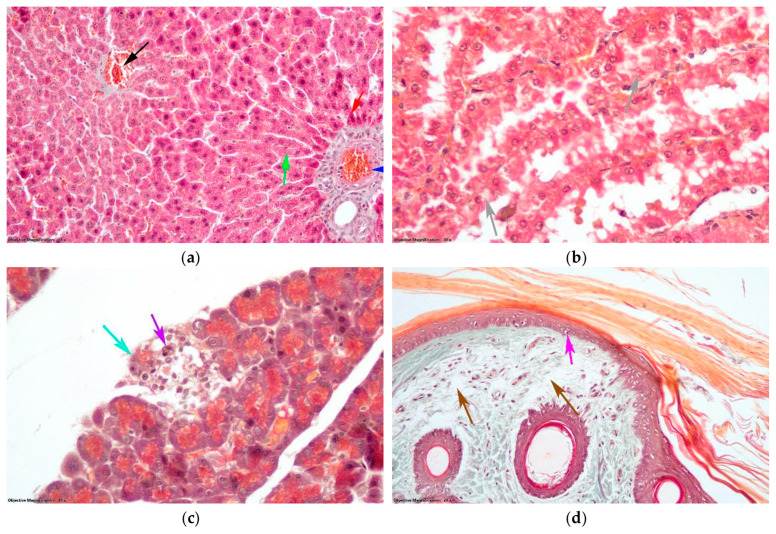
Histological changes (Goldner trichrome staining) in the group with D and thrombosis pretreated with LO at a dose of 100 mg/kg bw (DTL1 group): (**a**) Hepatic lobule: black arrow—centrilobular venule; blue arrow—portobiliary space; green arrow—hepatic steatosis with small droplets; red arrow—hepatocytes in cell death. (**b**) Nephron tubules: gray arrow—granulo-vacuolar degeneration. (**c**) Endocrine pancreas: turquoise arrow—endocrine cells from the islet of Langerhans; purple arrow—macrophage. (**d**) Dermis and epidermis: brown arrow—edema in papillary dermis; magenta arrow—pycnotic nuclei of keratinocytes.

In the DTL1 group, the liver had aspects similar to those of group D, with slight differences represented by the fact that small droplet hepatic steatosis was present in the outer half of the liver lobes, but there were fewer dead hepatocytes (Figure 11a). In the kidneys, there were areas where nephrocytes in the proximal tubule showed granulovacuolar degeneration (Figure 11b). In the pancreas, the necrosis of endocrine cells was observed in the islets of Langerhans (Figure 11c). In the skin, no thrombi or hemorrhages formed in the blood vessels. Moderate edema was observed in the hypodermis and derma. In very small areas, edema extended to the junction of the dermis and epidermis, and, in these areas, some keratinocyte nuclei in the deep layers were pycnotic (Figure 11d).

**Figure 12:**
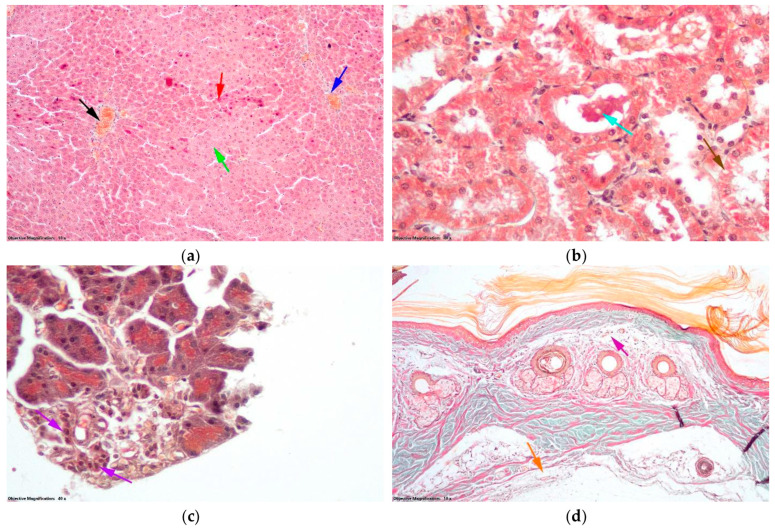
Histological changes (Goldner trichrome staining) in the group with D and thrombosis pretreated with LO at a dose of 200 mg/kg bw (DTL2 group): (**a**) Hepatic lobule: black arrow—centrilobular venule; blue arrow—portobiliary space; green arrow—hepatic steatosis with small droplets; red arrow—hepatocytes in cell death. (**b**) Nephron tubules: brown arrow—granulo-vacuolar degeneration; turquoise arrow—cellular detritus. (**c**) Endocrine pancreas: purple arrow—endocrine cells from the Langerhans islet. (**d**) Skin: orange arrow—edema in hypodermis; magenta arrow—edema in dermis.

In the DTL2 group in the liver, hepatocytes in the outer third of the liver lobe showed small droplet steatosis (Figure 12a) and the number of hepatocytes undergoing cell death was comparable to that in the group DTL1. In the kidneys, there were aspects of the granulo-vacuolar degeneration of nephrocytes in the proximal nephron tubule (Figure 12b) comparable in intensity and extent to those in the DTL1 group. Cellular detritus was also present in the lumens of tubules. Some cells showed cytoplasmic swelling in the endocrine pancreas, whereas others had pyknotic nuclei (Figure 12c). No thrombi or hemorrhages were observed on the skin of the blood vessels. Moderate edema was observed only in small areas of the hypodermis (Figure 12d).

## 4. Discussion

Our study demonstrated the dose-dependent antioxidant, anti-inflammatory, and organ-protective effects of lavender oil, with greater efficacy across all measured parameters at the higher dose (200 mg/kg bw, DTL2 group). Metabolic disorders related to diabetes mellitus affect the equilibrium between coagulation and fibrinolysis, resulting in a prothrombotic condition. Poor glycemic control and insulin resistance modify platelet function and the coagulation cascade, increasing the probability of thrombosis development [8,34]. Conversely, Han et al. [35] suggested that hypercoagulability is implicated in the development of diabetes mellitus. Impaired glucose metabolism and endothelial dysfunction are significant risk factors for the onset of T1DM and T2DM, and both are simultaneously linked to hypercoagulability [30]. Taken together, these factors play a significant role in the pathophysiology of diabetes mellitus. Furthermore, an inadequate antioxidant capacity in pancreatic β-cells leads to pancreatic cell failure in both T1DM and T2DM [36]. Linalool and linalyl acetate, primary constituents of lavender oil, demonstrated the potential to diminish oxidative stress, inflammation, and pathological conditions [37,38], which are leading contributors to numerous chronic conditions, including cardiovascular and neurodegenerative disorders [39,40]. Our study also demonstrated the therapeutic potential of lavender oil as an adjuvant for mitigating oxidative stress, inflammation, coagulation disturbances, and organ dysfunction associated with T1DM and thrombosis in a rat model.

Our findings outlined MDA, NOx, and TOS elevation in the D, T, and DT groups, highlighting the synergistic effects of hyperglycemia and thrombosis on oxidative stress (Table 1 and Figure 2). Various inflammatory mechanisms that influence platelets, the coagulation cascade, and the physiological functions of the vascular endothelium have been identified [6]. Our results are consistent with those of previous studies that have emphasized the role of oxidative stress as a precursor to endothelial dysfunction and thrombotic complications [41,42]. Pretreatment with LO significantly mitigated oxidative stress, with the DTL2 group showing reductions in MDA and TOS comparable to the control levels. Moreover, both groups pretreated with lavender oil (100 and 200 mg/kg bw) showed significant improvements in NOx levels (Table 1 and Figure 2). The reported results demonstrate the antioxidant properties of LO attributed to its main constituents, linalool, and linalyl acetate. Shin et al. demonstrated the antioxidant properties of linalyl acetate, with a reduction in MDA levels, a lipid peroxidation marker [43]. Our study showed statistically significantly decreased NOx levels at both lavender oil dosages. This underscores LO’s potential to alleviate nitro-oxidative stress, which has been linked to diabetic complications and vascular dysfunction [44]. Linalyl acetate was found to decrease NOx levels, indicating its potential to modulate NO production and attenuate oxidative stress [39]. Our results showed an improvement in total antioxidant capacity and thiol levels, further underscoring the efficacy of LO in restoring redox balance (Table 2 and Figure 3). Thiol levels, an antioxidant marker, were significantly improved in the DTL2 group (Figure 3). Elevated quantities of linalyl acetate, linalool, and flavonoids have been reported in lavender oil, indicating its antioxidant potential [45,46].

Inflammation, a key driver of thrombosis, diabetes mellitus, and diabetes mellitus complications, was demonstrated in our results by the elevated TNF-α, RANTES, and MCP-1 levels in the T, D, and DT groups (Table 3 and Figure 4). Our results indicate heightened inflammatory activity, which is consistent with the proinflammatory milieu observed in these conditions. TNF-α is a key mediator of insulin resistance and endothelial dysfunction in diabetes mellitus and thrombotic states [47,48]. In our study, lavender oil reduced the cytokine levels to near-control levels, particularly at higher doses (DTL2) (Figure 4). The anti-inflammatory effects of the major active compounds in lavender oil were previously evaluated with respect to the effects of linalool on lipopolysaccharide (LPS)-induced inflammation in BV2 microglial cells [49]. Li et al. [49] demonstrated that linalool inhibits the production of proinflammatory mediators, including TNF-α, interleukin (IL)-1β, NO, and prostaglandin E_2_ (PGE_2_). This inhibition was associated with the suppression of NF-κB activation, indicating that linalool exerts its anti-inflammatory effects by modulating the NF-κB signaling pathway [44,49]. We also observed a statistically significant reduction in MCP-1 levels in the LO pretreatment groups (Figure 4). MCP-1 is a chemokine critical for monocyte recruitment, and our results indicate LO’s potential to diminish vascular inflammation and leukocyte adhesion. Our results align with those of previous studies showing that the active compounds of lavender oil effectively reduced MCP-1 levels in inflammation in experimental rat models. Linalool treatment has been shown to decrease the levels of proinflammatory cytokines, such as MCP-1 and TNF-α, in a mouse model of lung inflammation induced by cigarette smoking [50]. Our assays showed that TNF-α and RANTES levels were significantly reduced at higher dosages of lavender oil compared to those in the DT groups (Figure 4). Low levels of TNF-α were reported in the group treated with lavender essential oil in an experimental mouse model [51]. The inhalation of lavender essential oil reduced depression-like behavior in an alcohol-withdrawn rat experimental model, with decreased hippocampal inflammatory factors, including IL-2, IL-6, IL-1β, and TNF-α [52].

Our measurements showed hyperglycemia and reduced C-peptide levels in the D and DT groups, underscoring the metabolic dysfunction characteristic of D (Table 4 and Figure 5). Pretreatment with LO resulted in glycemic control, with the DTL2 group achieving near-control levels, highlighting dose-dependent hypoglycemic potential (Figure 5). The partial restoration of C-peptide levels in the LO groups was outlined in our study (Figure 5), suggesting that LO may improve pancreatic β-cell function, likely owing to its antioxidant and anti-inflammatory effects on pancreatic cells. Lavender oil has been used as a therapeutic compound in traditional medicine to treat hyperglycemia [53]. Sebai et al. showed significantly reduced glucose levels in diabetic rats treated with lavender oil [54]. C-peptide is a biomarker for diabetes mellitus management and offers insights into endogenous insulin production. Our research showed a direct positive effect of lavender oil administration on C-peptide levels. In the scientific literature, there is a lack of data regarding the effects of the major compounds in lavender oil on C-peptide levels. Our findings, along with those of other studies that demonstrate the beneficial effects of natural compounds on C-peptide levels [55], underscore the potential of natural pharmaceuticals to improve endocrine pancreatic function.

The inadequate control of extracellular matrix remodeling by MMPs may lead to vascular problems in individuals with T1DM [56]. Dogaru et al. indicated that MMPs may modulate the synthesis of chemokines and cytokines, thereby contributing to innate immunity, inflammation, and angiogenesis [57]. Polymorphisms in genes encoding MMP-2 and MMP-9 have been linked to the development of microvascular complications in patients with diabetes mellitus, suggesting a genetic predisposition to altered MMP activity [58]. In our study, elevated levels of MMP-2 and MMP-9 in the DT group indicate enhanced extracellular matrix degradation and inflammation (Table 5 and Table 6). Pretreatment with LO reduced the MMP levels dose-dependently (Figure 6).

Shortened clotting time was observed in the T and DT groups compared to C; the results can be explained by the hypercoagulable state associated with thrombosis (Table 6). Pretreatment with LO extended these durations, suggesting antithrombotic properties. The bleeding time in DTL2 group showed no statistically significant difference than in the control group (Table 6). The coagulation and bleeding times in the DTL2 group were similar to those in the control group, suggesting a potential role in modulating platelet activation and aggregation (Table 6 and Figure 7). Our results align with those of our previous study on LO’s antithrombotic effects [24]. In addition, we demonstrated the adjuvant effect of LO pretreatment in rats with thrombosis treated with the traditional low-molecular-weight heparin fraxiparine [59].

The elevated levels of renal (creatinine and urea) and hepatic markers (ALT and AST) in the DT group indicate severe organ dysfunction exacerbated by the combined effects of T1DM and thrombosis (Table 7). Pretreatment with LO significantly improved these parameters, particularly at higher doses, indicating hepatoprotective and nephroprotective effects. Our findings are supported by studies demonstrating that LO’s active compounds have the capacity to attenuate oxidative stress and inflammation in renal and hepatic tissues. The hepatoprotective, nephroprotective, and pulmonary-protective effects of linalool are attributed to its anti-inflammatory properties [60]. Tamilmani et al. reported that linalool reduces lipid formation in the liver by suppressing de novo lipogenesis, enhancing fatty acid oxidation, and reducing oxidative stress by regulating the Sirt1/Akt/PPAR-α/AMPK and Nrf-2/HO-1 signaling pathways [61].

Our histopathological analyses revealed that LO reduced liver, kidney, and pancreatic tissue damage, with minimal pathological changes observed in the DTL2 group. The absence of thrombi in the skin vasculature of the LO-treated groups further emphasized LO’s antithrombotic properties. Our results are in line with those of previous research showing LO’s protective effects against oxidative and inflammatory damage in various organs.

## 5. Conclusions

Our study demonstrated dose-dependent antioxidant, anti-inflammatory, hypoglycemic, and organ-protective effects of *Lavandula angustifolia* oil in a rat model of T1DM and thrombosis. A dose of 200 mg/kg body weight was particularly effective in reducing oxidative stress markers and proinflammatory cytokines and improving metabolic parameters. The results reported in our manuscript suggest that lavender oil has potential as a complementary therapeutic agent for managing the inflammation and oxidative stress associated with T1DM and thrombosis.

## Figures and Tables

**Table 1:** Levels of oxidative stress markers in plasma by group.

Group	MDA, nmol/L	NOx, μmol/L	TOS, μmol H_2_O_2_ equiv./L
C	5.01 [4.84 to 5.12] {4.43 to 5.27}	24.31 [21.45 to 27.27] {17.28 to 29.66}	7.15 [6.49 to 7.66] {6.21 to 8.2}
T	6.26 [6.03 to 6.97] {5.79 to 8.26}	39.8 [36.74 to 41.68] {32.4 to 57.8}	21.85 [20.52 to 26.09] {19.88 to 26.97}
D	7.94 [7.59 to 8.43] {6.96 to 8.97}	76.8 [74.51 to 81.11] {69.45 to 90.68}	37.86 [35.36 to 39.35] {34.01 to 43.29}
DT	11.17 [10.32 to 11.8] {9.69 to 13.59}	92.47 [88.73 to 96.16] {84.32 to 104.32}	52.81 [49.01 to 55.01] {47.93 to 61.47}
DTL1	7.8 [7.64 to 8.04] {7.22 to 8.87}	47.11 [43.85 to 49.67] {40.67 to 54.69}	36.91 [34.4 to 38.51] {31.81 to 46.27}
DTL2	5.6 [5.43 to 5.94] {4.96 to 6.57}	40.19 [37.8 to 45.1] {32.77 to 50.08}	10.15 [9.18 to 11.72] {7.89 to 15.27}
*p*-value	<0.0001	<0.0001	<0.0001

Data are summarized as the median [Q1 to Q3] and {min to max}, where Q1 is the 25th percentile, Q3 is the 75th percentile, min is the minimum, and max is the maximum value. C = control group; T = thrombosis group; D = type 1 diabetes mellitus (T1DM) group; DT = T1DM and thrombosis group; DTL1 = T1DwM with 100 mg/kg bw lavender oil pretreatment; DTL2 = T1DM with 200 mg/kg bw lavender oil pretreatment; MDA = malondialdehyde; NOx = nitric oxide; TOS = total oxidative stress; and the *p*-value corresponds to the Kruskal–Wallis test.

**Table 2:** Plasma antioxidant capacity and thiol levels across groups.

Group	TAC, mmol Trolox/L	THIOL, μmol/L
C	1.09 [1.08 to 1.11] {1.06 to 1.14}	418.5 [406.25 to 446.25] {395 to 479}
D	0.97 [0.95 to 0.99] {0.89 to 1.02}	277 [260 to 292.5] {240 to 311}
T	0.75 [0.68 to 0.85] {0.57 to 0.89}	236.5 [220.25 to 257.25] {207 to 309}
DT	0.64 [0.58 to 0.76] {0.52 to 0.86}	217 [199.5 to 236] {176 to 279}
DTL1	0.87 [0.82 to 0.87] {0.77 to 0.91}	331 [315.5 to 340.5] {303 to 395}
DTL2	1.07 [1.05 to 1.08] {1 to 1.09}	437 [381 to 458.75] {339 to 489}
*p*-value	<0.0001	<0.0001

Data are summarized as the median [Q1 to Q3] and {min to max}, where Q1 is the 25th percentile, Q3 is the 75th percentile, min is the minimum, and max is the maximum value. C = control group; T = thrombosis group; D = type 1 diabetes mellitus (T1DM) group; DT = T1DM mellitus and thrombosis group; DTL1 = DT with 100 mg/kg bw lavender oil pretreatment; DTL2 = DT with 200 mg/kg bw lavender oil pretreatment; TAC = total antioxidant capacity; and the *p*-value corresponds to the Kruskal–Wallis test.

**Table 3:** Plasma inflammatory cytokines across groups.

Group	TNF-α, pg/mL	RANTES, pg/mL	MCP-1, ng/mL
C	38.07 [36.42 to 39.49] {28.87 to 42.59}	637.3 [555.03 to 732.35] {518.3 to 890.5}	1.15 [0.98 to 1.33] {0.85 to 1.95}
T	59.74 [54.15 to 69.36] {42.03 to 75.63}	984.2 [854.85 to 1200.9] {821.9 to 1396.1}	2.53 [2.24 to 2.63] {2.05 to 2.95}
D	73.11 [64.82 to 75.5] {52.24 to 79.33}	989.1 [885.9 to 1087.58] {823.5 to 1228.7}	2.33 [2.26 to 2.45] {1.95 to 2.85}
DT	80.17 [73.41 to 84.15] {69.95 to 93.94}	1398 [1243.43 to 1550.43] {1110.1 to 1746.2}	3.18 [2.8 to 3.38] {2.4 to 4.05}
DTL1	50.72 [44.51 to 52.44] {39.57 to 61.05}	1118.05 [907.95 to 1279.13] {834.4 to 1384.9}	2 [1.85 to 2.55] {1.55 to 2.8}
DTL2	39.28 [36.13 to 41.51] {29.86 to 44.29}	511.1 [453.8 to 622.15] {119.3 to 873.1}	1.88 [1.71 to 2.05] {1.35 to 2.2}
*p*-value	<0.0001	<0.0001	<0.0001

Data are summarized as the median [Q1 to Q3] and {min to max}, where Q1 is the 25th percentile, Q3 is the 75th percentile, min is the minimum, and max is the maximum value. C = control group; T = thrombosis group; D = type 1 diabetes (T1DM) mellitus group; DT = T1DM and thrombosis group; DTL1 = DT with 100 mg/kg bw lavender oil pretreatment; DTL2 = DT with 200 mg/kg bw lavender oil pretreatment; TNF-α = tumor necrosis factor α; RANTES = regulated on activation, normal T cell expressed and secreted; MCP-1 = monocyte chemoattractant protein 1; and the *p*-value corresponds to the Kruskal–Wallis test.

**Table 4:** Levels of glycemia and C-peptide by group.

Group	Glycemia, mg/dL	C-Peptide, pg/mL
C	98.94 [95.5 to 103.68] {89.91 to 105.06}	6574.85 [6285.15 to 6891.08] {6183.9 to 7484.7}
T	116.34 [108.58 to 122.49] {99.34 to 143.22}	4700.05 [4357.2 to 5109.98] {4100.6 to 5724.5}
D	290.22 [286.71 to 295.24] {272.76 to 304}	1637.35 [1249.05 to 1816.95] {874.2 to 2649.6}
DT	317.34 [308.71 to 326.16] {305.45 to 333.46}	546.5 [427.95 to 639.9] {302.8 to 878.4}
DTL1	264.67 [249.47 to 270.99] {237.4 to 276.8}	4084.95 [3958.75 to 4431.5] {3792.7 to 4688.3}
DTL2	219.81 [200.54 to 231.83] {190.99 to 258.61}	4618.05 [4502.38 to 4812.18] {4277.4 to 5441.6}
*p*-value	<0.0001	<0.0001

Data are summarized as the median [Q1 to Q3] and {min to max}, where Q1 is the 25th percentile, Q3 is the 75th percentile, min is the minimum, and max is the maximum value. C = control group; T = thrombosis group; D = type 1 diabetes mellitus (T1DM) group; DT = T1DM and thrombosis group; DTL1 = DT with 100 mg/kg bw lavender oil pretreatment; DTL2 = DT with 200 mg/kg bw lavender oil pretreatment; and the *p*-value corresponds to the Kruskal–Wallis test.

**Table 5:** Plasma levels of MMP-2 and MMP-9 across groups.

Group	MMP-2, ng/mL	MMP-9, ng/mL
C	85.25 [80.37 to 92.78] {64.6 to 99.65}	21.23 [19.68 to 23.14] {17.9 to 26.6}
T	147.85 [134.57 to 168.92] {108.36 to 188.95}	23.25 [20.75 to 24.58] {19.1 to 27.8}
D	189.02 [174.68 to 206.02] {119.2 to 241.68}	43.3 [40.53 to 45.93] {39.2 to 48}
DT	264.17 [227.76 to 273.87] {204.17 to 343.87}	43.65 [40.71 to 45.24] {36.9 to 48.6}
DTL1	189.06 [147.73 to 211.06] {132.86 to 303.83}	37.43 [35.38 to 38.69] {31.15 to 42.2}
DTL2	158.13 [140.08 to 170.65] {115.81 to 193.06}	26.8 [23.48 to 32.48] {22.05 to 39.45}
*p*-value	<0.0001	<0.0001

Data are summarized as the median [Q1 to Q3] and {min to max}, where Q1 is the 25th percentile, Q3 is the 75th percentile, min is the minimum, and max is the maximum value. C = control group; T = thrombosis group; D = type 1 diabetes (T1DM) mellitus group; DT = T1DM and thrombosis group; DTL1 = DT with 100 mg/kg bw lavender oil pretreatment; DTL2 = DT with lavender oil 200 mg/kg bw lavender oil pretreatment; MMP-2 = metalloproteinase 2; MMP-9 = metalloproteinase 9; and the *p*-value corresponds to the Kruskal–Wallis test.

**Table 6:** Variation in bleeding and clotting time across groups.

Group	Bleeding Time, s	Clotting Time, s
C	170 [161.25 to 173.5] {153 to 180}	137 [131.5 to 145.5] {128 to 150}
T	73 [66.5 to 78.25] {61 to 94}	55 [48.75 to 57.5] {47 to 61}
D	144.5 [140.5 to 150] {137 to 159}	124.5 [120 to 129] {117 to 133}
DT	61 [59 to 65.5] {54 to 71}	45 [44 to 47.5] {41 to 53}
DTL1	104 [98.75 to 106.5] {94 to 113}	91.5 [88 to 95.5] {87 to 101}
DTL2	120 [114.5 to 130] {109 to 146}	106 [104.25 to 108.75] {99 to 115}

Data are summarized as the median [Q1 to Q3] and {min to max}, where Q1 is the 25th percentile, Q3 is the 75th percentile, min is the minimum, and max is the maximum value. C = control group; T = thrombosis group; D = type 1 diabetes mellitus (T1DM) group; DT = T1DM and thrombosis group; DTL1 = DT with 100 mg/kg bw lavender oil pretreatment; and DTL2 = DT with 200 mg/kg bw lavender oil pretreatment.

**Table 7:** Plasma levels of creatinine, urea, ALT, and AST across groups.

Group	Creatinine, mg/dL	Urea, mg/dL	ALT, UI	AST, UI
C	0.71 [0.67 to 0.73] {0.62 to 0.78}	39.5 [38.25 to 42.25] {35 to 46}	34.5 [32.25 to 38] {26 to 40}	40.5 [39 to 43.75] {34 to 46}
T	0.96 [0.84 to 1.02] {0.74 to 1.36}	53.5 [47.5 to 59.75] ^b1^ {45 to 82}	68.5 [58.5 to 73.25] {54 to 79}	62.5 [57.75 to 67.25] {49 to 80}
D	1.31 [1.21 to 1.67] ^a1^ {1.09 to 1.9}	73 [59 to 76.75] ^b2^ {53 to 82}	152 [136.75 to 156.75] ^c1^ {117 to 164}	122.5 [118 to 126.75] ^d1,d2^ {107 to 139}
DT	1.89 [1.86 to 2] ^a2,a3^ {1.78 to 2.15}	87 [84.25 to 89.75] ^b3,b4^ {75 to 93}	176.5 [167.25 to 185] ^c2,c3^ {159 to 193}	140.5 [135.25 to 148] ^d3,d4^ {119 to 154}
DTL1	1.03 [0.88 to 1.2] ^a4^ {0.81 to 1.39}	50.5 [48 to 59] ^b5^ {46 to 64}	135 [132.25 to 142.75] ^c4^ {132 to 161}	86 [84 to 98] ^d5^ {80 to 116}
DTL2	0.75 [0.71 to 0.84] ^a5,a6^ {0.67 to 0.92}	49 [46.5 to 50.75] ^b6^ {43 to 58}	64 [60.5 to 75.5] ^c5^ {52 to 84}	65 [59 to 69.75] ^d6,d7^ {51 to 86}

Data are summarized as the median [Q1 to Q3] and {min to max}, where Q1 is the 25th percentile, Q3 is the 75th percentile, min is the minimum, and max is the maximum value. C = control group; T = thrombosis group; D = type 1 diabetes mellitus (T1DM) group; DT = T1DM and thrombosis; DTL1 = DT with 100 mg/kg bw lavender oil pretreatment; DTL2 = DT with 200 mg/kg bw lavender oil pretreatment; ALT = alanine aminotransferase; and AST = aspartate aminotransferase. Post hoc analysis: ^a1^ 0.0001 C vs. D; ^a2^ <0.0001 C vs. DT; ^a3^ 0.0086 T vs. DT; ^a4^ 0.0256 C vs. DTL1; ^a5^ 0.0036 D vs. DTL2; ^a6^ <0.0001 DT vs. DTL2; ^b1^ 0.0279 C vs. T; ^b2^ 0.0001 C vs. D; ^b3^ <0.0001 C vs. DT; ^b4^ 0.0262 T vs. DT; ^b5^ 0.0057 DT vs. DTL1; ^b6^ 0.0004 DT vs. DTL2; ^c1^ <0.0001 C vs. D; ^c2^ <0.0001 C vs. DT; ^c3^ 0.0002 T vs. DT; ^c4^ 0.0003 C vs. DTL1; ^c5^ 0.0002 DT vs. DTL2; ^d1^ <0.0001 C vs. D; ^d2^ 0.0114 T vs. D; ^d3^ <0.0001 C vs. DT; ^d4^ 0.0002 T vs. DT; ^d5^ 0.0022 C vs. DTL1; ^d6^ 0.0220 D vs. DTL2; and ^d7^ 0.0004 DT vs. DTL2.

## Data Availability

The raw data analyzed in this study are part of a Ph.D. study and can be obtained upon reasonable request from Valeriu Mihai But (but.valeriumihai@elearn.umfcluj.ro).
